# Emissions from Gas Distribution and Postmeter Leaks May Drive Seasonal and Diurnal Trends in Methane Emissions in Baltimore

**DOI:** 10.1021/acs.est.6c03452

**Published:** 2026-09-02

**Authors:** Helen C. R. Kenion, Kenneth J. Davis, Natasha L. Miles

**Affiliations:** † Department of Meteorology and Atmospheric Science, The Pennsylvania State University, University Park, Pennsylvania 16802, United States; ‡ Earth and Environmental Systems Institute, The Pennsylvania State University, University Park, Pennsylvania 16802, United States

**Keywords:** urban, greenhouse gas, emissions inventory, pipeline leaks, atmospheric emissions monitoring

## Abstract

We estimated diurnal and seasonal trends in methane (CH_4_) emissions and evaluated their relationships with trends in natural gas usage data in Baltimore, MD, USA. We used Monin-Obukhov Similarity Theory for flux estimation at urban, suburban, and rural sites using several years of tower mole fraction measurements and compared flux estimates to hourly natural gas usage data for one year at the urban sites. A diurnal pattern with larger daytime emissions is present at all sites and lags a similar diurnal pattern in natural gas usage at sites where usage data is available, suggesting the diurnal trends are related to postmeter emissions. The estimates show a seasonal trend and higher emissions at the urban sites which we expect is related to natural gas distribution pipeline leaks and leak maintenance schedules. These seasonal trends are absent at the suburban sites which are in areas where pipelines have likely been upgraded. Our results reinforce that postmeter gas emissions are likely an important source of urban CH_4_ emissions. We expect that gas pipeline leak maintenance schedules play an important role driving seasonal CH_4_ emissions trends, and differences in pipe material can cause within-city variability in CH_4_ emissions magnitudes and trends.

## Graphical abstract

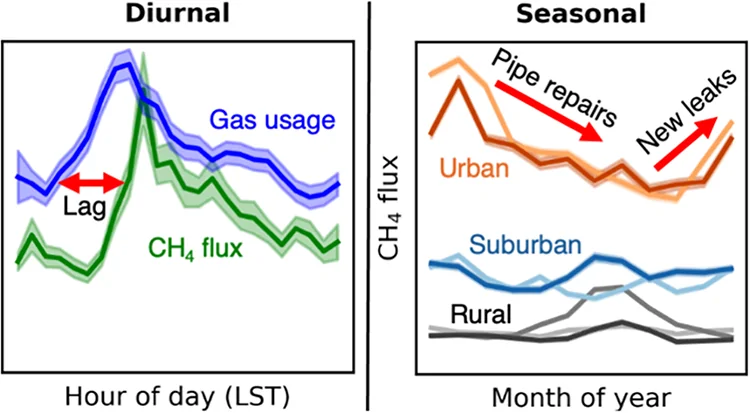

## Introduction

Accurate emissions tracking is essential to implement effective greenhouse gas (GHG) emissions mitigation strategies. Urban areas account for more than half of anthropogenic GHG emissions,^1^ making accurate accounting especially important in these complex environments. To monitor urban emissions and evaluate the effectiveness of emissions reduction actions, decisionmakers typically use human activity-based estimates of emissions (inventories) because these can be created for many cities based on reported data.^2^ Atmospheric emissions monitoring methods, while not available in every city, are useful to provide independent emissions estimates to evaluate and improve inventories.^3,4^

Methane (CH_4_) is a crucial target for emissions reduction but is especially challenging to quantify using inventory methods. Because CH_4_ has a high global warming potential and relatively short lifetime,^5^ CH_4_ emissions reduction can make a large climate change mitigation impact on a relatively short time scale. Urban CH_4_ emissions sources include landfills, wastewater, and natural gas leaks, which by nature are not as correlated with human activity as, for example, carbon dioxide (CO_2_) emissions from traffic or energy production. These source types make estimating urban CH_4_ emissions from activity data challenging, making atmospheric emissions estimates even more essential for inventory development and evaluation.^6–8^

Many atmospheric studies have found evidence linking urban methane emissions with fugitive natural gas leaks. Results from urban studies in several cities have suggested that missing fossil fuel sources contribute to inventory underestimation of methane,^8–10^ and some studies have identified leaks in natural gas distribution systems as methane sources using mobile measurements or measurements near pipelines.^7,11–15^ Seasonal trends with higher winter emissions or emissions correlating with natural gas usage have been identified in several cities,^6,9,16–18^ suggesting fugitive emissions at the end use (postmeter emissions). Studies taking CH_4_ measurements of or near homes have reported evidence that appliances including cooking appliances, water heaters, and furnaces are important sources of fugitive CH_4_ emissions.^12,19–22^ Based on measurements of CH_4_ at several representative homes, Fischer et al.^19^ calculated that emissions of natural gas in California homes may make up approximately 15% of the state’s CH_4_ emissions from natural gas.

Biogenic CH_4_ sources also play an important role in urban areas, including landfills and wastewater treatment plants.^7–9,14,23^ Fugitive emissions from wastewater collection systems have also been reported, with varying significance relative to fugitive natural gas emissions.^24–26^

Atmospheric emissions estimates with high spatial and temporal resolutions could help disentangle urban CH_4_ sources. For example, eddy covariance can be used to measure GHG emissions at up to half-hourly temporal resolution^27^ on a local-scale, with a meteorology-dependent footprint extending approximately 10 times the height of the measurement from the point of measurement.^28^ This spatial and temporal resolution can make it easier to link emissions patterns to individual emissions sources or source sectors^29,30^ in complex urban environments with several GHG sources. Most urban eddy covariance GHG studies to date have focused on CO_2_,^29,31–34^ and eddy covariance measurements of CH_4_ flux in urban environments are relatively rare.^30,35–39^ Alternatively, local-scale, hourly GHG fluxes can be approximated from tower-based mole fraction measurements using Monin-Obukhov similarity theory (MOST).^40,41^ This method takes advantage of valuable data available from existing urban tower networks designed for inverse emissions estimation^42–45^ while benefiting from temporal and spatial resolution similar to eddy covariance. MOST estimates are complementary to tower-based inversion estimates, which are most useful for total-city emissions estimation. Tower-based inversions have been reported to have increasing uncertainty with increasing spatial resolution^46^ or a high dependence of the spatial structure on the prior.^16^ At high resolutions, there are few options for coevaluation of inversion estimates or inventories, and MOST^41^ and eddy covariance^31,32,47^ can fill that need.

In this study, we use MOST’s flux-variance relationship to estimate local-scale CH_4_ fluxes using mole fraction measurements from seven tower sites in and near Baltimore, MD, USA. These sites include two urban, two suburban, and three rural sites.^42^ We compare the estimated CH_4_ fluxes at the two urban sites to natural gas usage data and compare all but one (rural) site to a gridded regional CH_4_ inverse emissions estimate by Karion et al.^16^

## Methods and Data

### Measurement Sites and Instrumentation

We used CH_4_ dry mole fraction measured at two urban sites in Baltimore, MD, two suburban sites near Baltimore, and three rural sites in the Washington, DC. and Baltimore region^48^ to estimate local-scale CH_4_ fluxes surrounding each site (Figures 1, 2, and Table 1). These sites are part of the National Institute of Standards and Technology’s (NIST) Northeast Corridor GHG mole fraction monitoring network.^42^ The measurements are taken at a sampling frequency of approximately 2–3 s with wavelength-scanned cavity ring down spectroscopic (CRDS) instruments (Picarro G2301 and G2401). At these sites, the Picarro cycles through two inlets, measuring 40 min at the top inlet, then 20 min at the lower inlet. We used the standard deviation of each period of continuous sampling to calculate a flux estimate. A description of data processing for calculating the standard deviation is provided in the Supporting Information.

**Figure 1:**
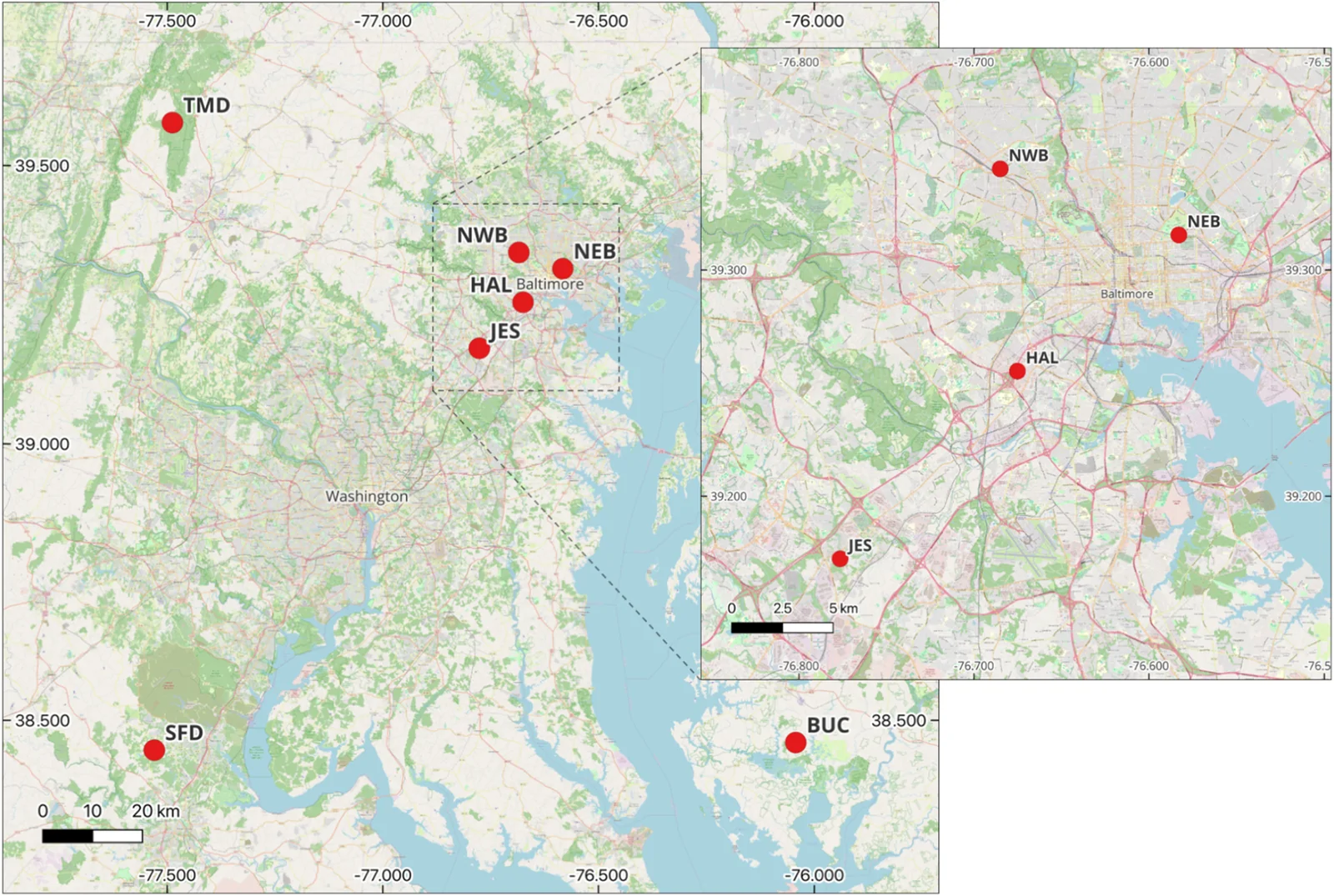
CH_4_ measuring site locations shown by red dots. More site information listed in Table 1. Basemap source: OpenStreetMap.

**Figure 2:**
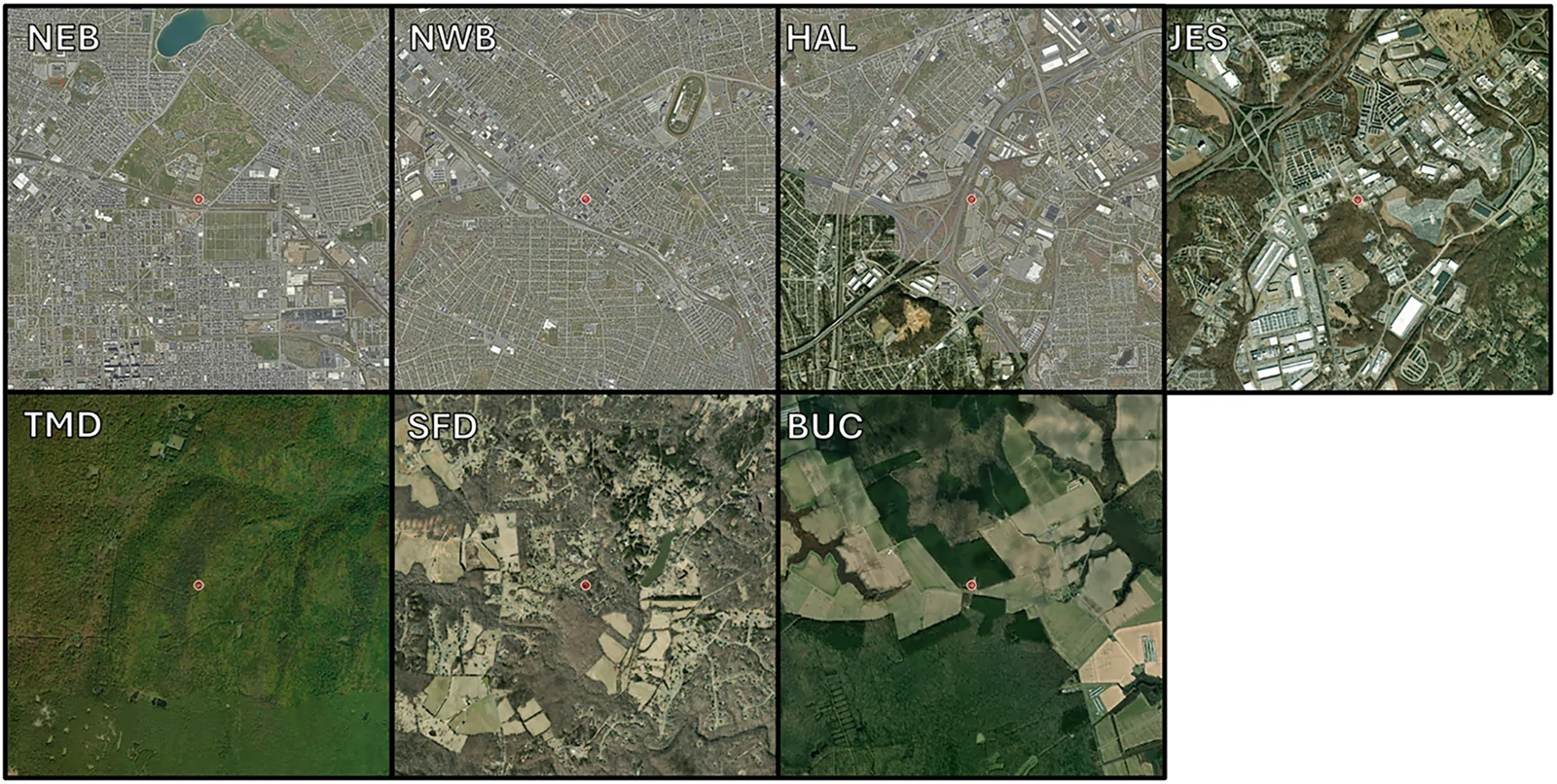
4 km by 4 km maps centered around each monitoring site. The site is indicated by a red and white circle. Some color changes may indicate temporal differences in imaging rather than spatial differences. Satellite image sources: Esri, DigitalGlobe, GeoEye, i-cubed, USDA FSA, USGS, AEX, Getmapping, Aerogrid, IGN, IGP, swisstopo, and the GIS User Community.

**Table 1:** List of Mole Fraction Monitoring Sites Used^42^

site name	type	location	latitude	longitude	inlet height used	temporal extent
NEB	Urban	NE Baltimore, MD	39.3154	–76.5830	50m AGL	09/2016–12/2024
NWB	Urban	NW Baltimore, MD	39.3445	–76.6851	27m AGL	09/2016–12/2024
HAL	Suburban	Halethorpe, MD	39.2552	–76.6753	29m AGL	10/2015–12/2024
JES	Suburban	Jessup, MD	39.1723	–76.7765	49m AGL	05/2016–12/2024
TMD	Rural	Thurmont, MD	39.5768	–77.4881	49m AGL	05/2017–12/2024
SFD	Rural	Stafford, VA	38.4459	–77.5300	50m AGL	07/2017–12/2024
BUC	Rural	Bucktown, MD	38.4597	–76.0430	46m AGL	01/2015–12/2024

### Local-Scale Flux Estimation

We used the MOST flux-variance relationship to estimate local-scale CH_4_ fluxes using the standard deviation of CH_4_ mole fraction measurements as described by Kenion et al.^40^ When using Automated Surface Observing System (ASOS) station (weather station) data^49^ to estimate the meteorological variables required for flux estimation,^40^ we used wind, humidity, pressure, and cloud cover measurements from the nearest ASOS station unless restricted by data availability. In some cases, data from a combination of sites were used if, for example, the nearest site had all variables needed except pressure. We kept constant the weather station(s) whose data we used for flux estimation at each mole fraction monitoring site.

We estimated fluxes for each continuous mole fraction measuring period using the variables calculated from the ASOS station measurements that were nearest in time to the middle of the mole fraction sampling period. For data analysis, we produced hourly fluxes from these estimates by taking hourly means (typically including only one data point) where the timestamp represents the beginning of the averaging period and the middle point of the flux estimate’s mole fraction sampling time is used to determine the averaging period in which the flux estimate is included.

We divide all flux estimates at each site by a constant-in-time correction factor to correct for systematic overestimation of GHG fluxes by the flux-variance method. Kenion et al.^40^ identified overestimation by the flux-variance method and speculated that the factor of overestimation may be site-specific. This has been tested at two sites, reporting a ratio of CO_2_ fluxes estimated using the flux-variance method and colocated eddy covariance CO_2_ flux measurements of 1.29 at an urban site^40^ and 1.14 at a suburban site.^41^ We do not have the information needed to calculate site-specific correction factors at these towers. We divided all hourly flux estimates by 1.29, the ratio reported by Kenion et al.^40^ In this analysis, we focus on results that are less sensitive to this uncertainty including temporal trends in emissions and site-by-site differences in magnitude that are much larger than 15%.

We applied friction velocity filtering to remove flux estimates during stable nighttime conditions when our estimates may not be representative of the surface flux. At each site, we inspected the hourly nighttime flux as a function of friction velocity to identify a critical u_*_ threshold (u_*crit_) below which the flux decreases with decreasing u_*_,^50^ where nighttime is defined as a solar zenith angle below 90°. We identified a u_*crit_ of 0.08 m/s at HAL and BUC and a u_*crit_ of 0.13 m/s at the remaining sites. Because the ASOS wind measurements are discretized (rounded to the nearest knot with a detection threshold of 3 knots), we do not have the resolution to compute low friction velocities. With higher wind speed resolution, a more precise u_*crit_ could be identified. We removed all nighttime flux estimates whose associated u_*_ fell below the site-specific u_*crit_.

We filtered out wind directions from which measurements could be affected by point source plume interference. Plumes from sources outside of the flux footprint complicate interpretation of estimated fluxes and violate the underlying assumptions of MOST. We identified CH_4_ point sources reported by the U.S. Environmental Protection Agency^51^ within 25 km of each mole fraction monitoring site. If there was a large fraction of high estimated fluxes over a narrow band of wind directions that matched the direction of a large-emitting point source, we excluded all data from wind directions within 30° of direction of the point source from our analysis. We chose these conservative distance and wind direction thresholds based on visual inspection of the data given the location of known point sources. We identified a high frequency of relatively large estimated CH_4_ emissions at HAL from the direction of the Quarantine Road landfill approximately 11 km away, so we excluded flux estimates from wind directions 86.8°–146.8°. We also identified a high frequency of large CH_4_ emissions estimates at SFD in the direction of the Stafford County landfill approximately 13 km away and excluded flux estimates from wind directions 95°–155°. We did not identify point source interference at the other sites.

We applied standard deviation filtering to the estimated fluxes at each site, removing all hourly flux estimates that exceeded 10 standard deviations from the mean using a 30-day moving window. We conducted visual inspections of filtered data to ensure this filter did not introduce bias.

### Natural Gas Usage Data

We compared trends in our estimated CH_4_ fluxes at NEB and NWB to trends in natural gas usage data supplied by the local gas distribution company.^52^ This usage data is averaged over blocks of 15 or more addresses to maintain anonymity, which we refer to as 15-address blocks for simplicity. Surrounding each urban tower site, we averaged usage data for all blocks that included any address within 1km of the measurement site. Hourly gas usage data is only available for service points that were equipped with advanced metering infrastructure. This analysis includes data from 3649 total service points which, based on the number of addresses in our domain, we estimate represents at least 44% of service points in our domain. Usage data is only available for the year 2023. The suburban and rural sites are not included in this analysis because they are outside of the spatial domain of the usage data provided by the gas company. Prior to our analysis, we removed 1 h of anomalously high gas usage at NEB (July 16th 04:00 with an area averaged reported usage of 601 therms).

### Comparison to Karion et al. (2023) Inversion Prior and Posterior

We compared our estimated fluxes to Karion et al.’s^16^ gridded, monthly CH_4_ flux estimate inversion posterior^53^ and to the gridded, time-invariant prior (bottom-up emissions estimate)^53^ used in Karion et al.’s inversion estimate. Karion et al.’s estimates are gridded at 0.01° by 0.01° for the Baltimore/Washington region. The inversion estimate used a five-year record of mole fraction measurements from several tower sites in the NIST Northeast Corridor network, including the sites we use in this study. For each measurement site, we took the average of all prior and posterior grid cells whose center was within 1.8 km of the measurement site location for an approximate spatial match. We chose 1.8 km under the simplified assumption that the footprint extends approximately 1km from the point of measurement. Considering this assumption and the grid cell size of approximately 1.1 km by 1.1 km, the furthest a grid cell center point could be from the measurement site while the grid cell still touches the 1km footprint radius is approximately 1.8 km. Because the trajectory of the emissions from natural gas may include more interactions than are represented in a footprint model (e.g., postmeter emissions that interact with building ventilation before reaching outdoors), and because of the spatial resolution of the prior and posterior, we did not use a footprint model for this comparison. To understand the extent our simplified footprint assumption affects our results, we also performed the comparison assuming a footprint extent of 2 km, provided in the Supporting Information. The inversion posterior includes 60 ensembles, using different configurations of realistic meteorological fields, background conditions, and priors. This includes five different priors, each based on the same initial emissions map but with varying magnitudes of emissions from the natural gas, waste, and wetland sectors. We compared our flux estimates to the mean of the 60 posterior ensembles and to the mean of the 5 priors.

### Analysis

We evaluated average seasonal trends and average daily cycles of estimated CH_4_ emissions and natural gas usage. To calculate seasonal trends, we calculated an average value for each month of the year. To avoid potential bias due to any uneven spread of missing data by time of day, for each month, we bin averaged the hourly data for the entire month by hour of day, resulting in 24 average hours, then took the average of those 24 points. To quantify the uncertainty, we used statistical bootstrapping to estimate the standard error. For each month of the year at each site, we took 1000 random samples from the overall hourly data set. Each sample matched the number of data points for each hour of the day for the given month at the given site. Then, we took the mean of the average day for each random sample, producing 1000 means. We then calculated the standard deviation of the means, producing the standard error. To calculate average daily cycles, we calculated the average and standard error of all values for each hour of the day.

We calculated correlation coefficients between the natural gas usage data and the estimated CH_4_ emissions surrounding NEB and NWB. At each site, we calculated the Spearman r for the hourly data without additional averaging, which we refer to as the “hourly” correlation coefficient. We calculated the Spearman r between natural gas usage data and the estimated CH_4_ emissions using the 24 data points in the average daily cycles and refer to this as the “diurnal” correlation. We calculate the Spearman r between the natural gas usage and CH_4_ emissions using the 12 monthly averages (weighed by hour of day as described above) and refer to this as the “monthly” correlation coefficient.

## Results and Discussion

### Seasonal Trends

#### CH_4_ Emissions by Site

The estimated flux magnitudes and seasonal trends differ by site type (urban, suburban, and rural). The estimated flux magnitudes at the urban sites are approximately twice those of the two suburban sites, whose estimated flux magnitudes are approximately twice those of the rural sites, besides BUC in July and August (Figure 3).

**Figure 3:**
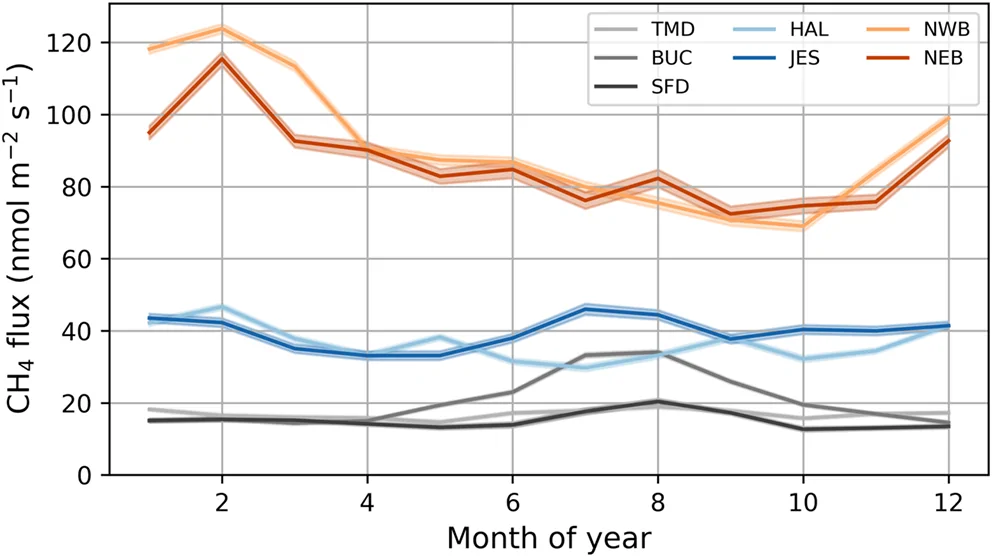
Seasonal trends of estimated CH_4_ flux at each measurement site. Temporal extents (6 to 9 years, depending on site) match those listed in Table 1. The shaded region represents one standard error calculated using statistical bootstrapping. Seasonal trends at rural sites (TMD, BUC, SFD) are shown in gray, suburban sites in blue (HAL, JES), and urban sites in orange (NWB, NEB).

The difference in the flux magnitudes between urban and suburban sites in Baltimore could be partially explained by natural gas pipeline material. The natural gas pipelines in Baltimore’s suburban areas have been upgraded from cast iron pipes to plastic pipes, which are more flexible and less prone to leaks, while the urban center of Baltimore has not been upgraded yet (personal communications, local gas company representatives). Von Fischer et al.^15^ reported CH_4_ emissions approximately 25 times larger in cities with corrosion-prone pipeline materials compared to cities with newer pipes, and several other studies have reported tentative links between pipeline materials and CH_4_ emissions.^7,11,54,55^ This could explain why the estimated fluxes at NWB and NEB, located in urban areas where we expect cast iron pipelines, are larger in magnitude than the estimated fluxes at HAL and JES, located in suburban areas where we expect plastic pipelines.

Estimated fluxes at the two urban sites, NEB and NWB, follow a near-identical seasonal trend, with increasing emissions beginning after October reaching a peak in February before decreasing to the October low (Figure 3). This trend is absent at both suburban sites, JES and HAL, and at the rural sites TMD and SFD, all of which have relatively flat seasonal trends. BUC has a unique seasonal trend with increased summer emissions.

Differences in the shape of the seasonal trends in estimated CH_4_ fluxes between site types could also be explained by differences in natural gas pipeline materials. In winter, the depth to which the ground is frozen (freeze line) changes, causing pipes to shift. The shifting freeze line causes leaks in rigid cast iron pipelines, while flexible plastic pipelines are more resilient. These leaks form faster than they can be identified and repaired, resulting in an accumulation of leaks until repairs are completed by the local gas company. It typically takes until fall, shortly before the cold season begins again, for all the leak repairs to be completed (personal communications, local gas company representatives). This pattern is consistent with the seasonal trend in CH_4_ emissions present at NWB and NEB, the two urban sites, and could explain why the seasonal trends at the two suburban sites, JES and HAL, are relatively flat as we expect that pipelines in the suburban areas have been upgraded to plastic while those in the urban areas are likely still cast iron (personal communication, local gas company representatives). It would also explain why the seasonal trend present at the urban sites does not match those of the rural sites, where we expect less natural gas infrastructure.

Postmeter emissions may also contribute to differences in emissions magnitude between sites. We expect that the denser urban regions have more natural gas infrastructure and appliances than the suburban regions, followed by the rural areas. However, there is no evidence that seasonal trends of postmeter emissions should differ between urban and suburban areas.

Unlike the urban sites whose emissions trends may be driven by natural gas emissions, the seasonal trend at BUC could be explained by wetland emissions. Karion et al.^42^ reported that over 40% of the land cover within 5 km of BUC is wetlands, and wetland CH_4_ emissions follow seasonal trends with higher emissions in summer.^56,57^ The higher summer emissions observed at BUC are consistent with the larger summertime CH_4_ enhancements observed by Karion et al.^16^

#### Comparison to Natural Gas Usage Data

Both the natural gas usage data and the estimated CH_4_ fluxes have higher winter emissions in 2023, but the overall month-to-month trends differ (Figure 4). Surrounding both sites, the January and December natural gas usage is over five times higher than the June-August usage. The usage starts high in January, decreasing slowly until an abrupt decrease from March to April, then a slow decrease to a low in July or August, a slow increase until an abrupt increase from October to November, and a slow increase back to a high in December. The 2023 flux estimates around NWB are also highest in winter, but they have a slower and more jagged decrease to a low in the fall (September) rather than in summer. The 2023 flux estimates surrounding NEB oscillate more and do not have a clear January/February high but follow a similar general pattern as the NWB flux estimates, with a low in September before an increase toward the end of the year. The fractional difference between the high and low months in the estimated fluxes is not as dramatic as that of the natural gas usage, but still substantial at around a factor of 2. The correlation between the monthly averages at NWB is statistically significant and relatively high, but at NEB, it is not statistically significant (Table 2).

**Figure 4:**
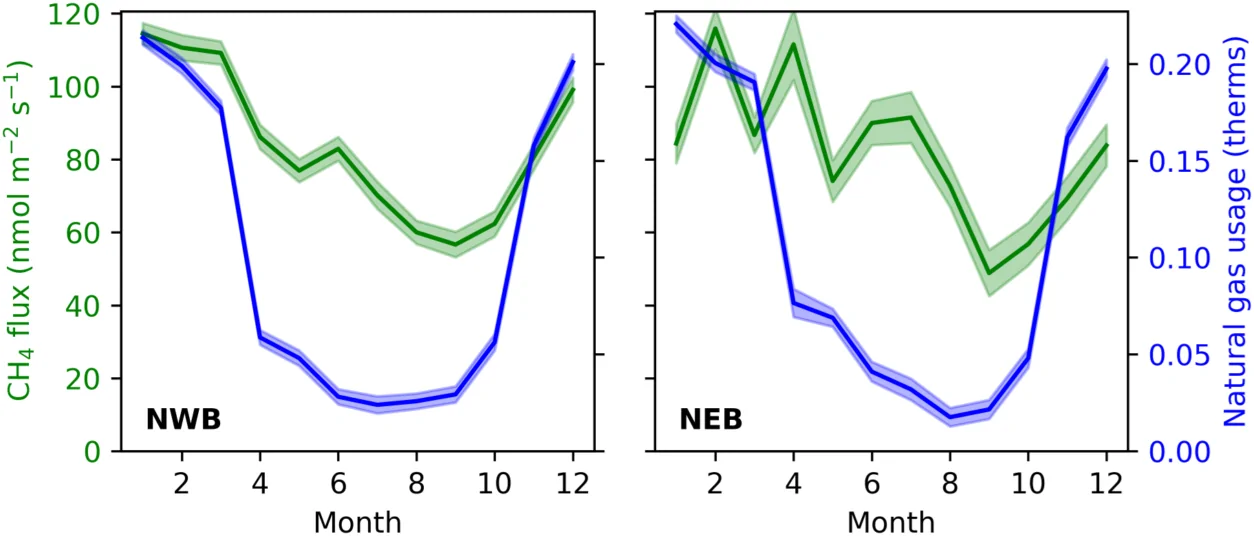
Monthly averaged estimated CH_4_ fluxes (green) and natural gas usage (blue) at sites NWB (left) and NEB (right) for the year 2023. The shaded region represents one standard error calculated using statistical bootstrapping.

**Table 2:** Spearman *r* Values between Natural Gas Usage Data and Flux Estimates at NEB and NWB for the Year 2023^a^

	no lag			1 h lag		2 h lag		3 h lag	
	hourly	diurnal	monthly	hourly	diurnal	hourly	diurnal	hourly	diurnal
NWB	0.30^b^	0.62^b^	0.81^b^	0.32^b^	0.77^b^	0.33^b^	0.80^b^	0.33^b^	0.80^b^
NEB	0.074^b^	0.49^b^	0.38	0.089^b^	0.74^b^	0.099^b^	0.88^b^	0.10^b^	0.90^b^

a We also present hourly and diurnal correlations with the natural gas usage data lagged by 1–3 h.

b Indicates the correlation is statistically significant at 95% confidence. A table with Pearson *r* values can be found in the Supporting Information.

The cause of the differences in trends and correlation of estimated CH_4_ fluxes with usage data between NEB and NWB at monthly time scale is unknown. The 2023 month-to-month trend at NEB appears noisier than that at NWB, but the number of hourly data points for estimated CH_4_ flux for each month of the year is similar, except for the month of April. Excluding April, the mean number of hourly data points used to calculate each monthly weighted average is 426 at NEB and 494 at NWB, and for April, it is 175 at NEB and 529 at NWB. Because we expect that the seasonal trend is affected by both pipeline maintenance schedules and postmeter emissions, we do not necessarily expect high correlation between the seasonal trends of gas usage and CH_4_ emissions. Some of the correlation at NWB could be associated with postmeter emissions, while some could be because of increased pipeline leaks during winter because of freeze–thaw cycles that coincide with increased gas usage. The low correlation at NEB could potentially be because the month-to-month trend in estimated CH_4_ emissions may be driven by pipeline maintenance schedules that are variable and do not always correlate with gas usage.

### Diurnal Trends

#### Diurnal Trends by Site

The diurnal trend in estimated CH_4_ fluxes is similar across all sites (Figure 5, left). All sites have low, relatively flat estimated emissions at night (approximately hours 19–5 LST) followed by a morning increase beginning around hour 4–6 LST to a peak around 8–10 LST. Emissions stay relatively constant or decrease slowly for a few hours before beginning to decrease in the afternoon (around 12–14 LST) back to the nighttime low. In winter, the urban sites (NEB and NWB) have a larger diurnal amplitude. There is not a clear seasonal pattern in diurnal amplitude at the suburban and rural sites. All sites have an earlier morning increase and later evening decrease in summer compared to winter (Figure 5).

**Figure 5:**
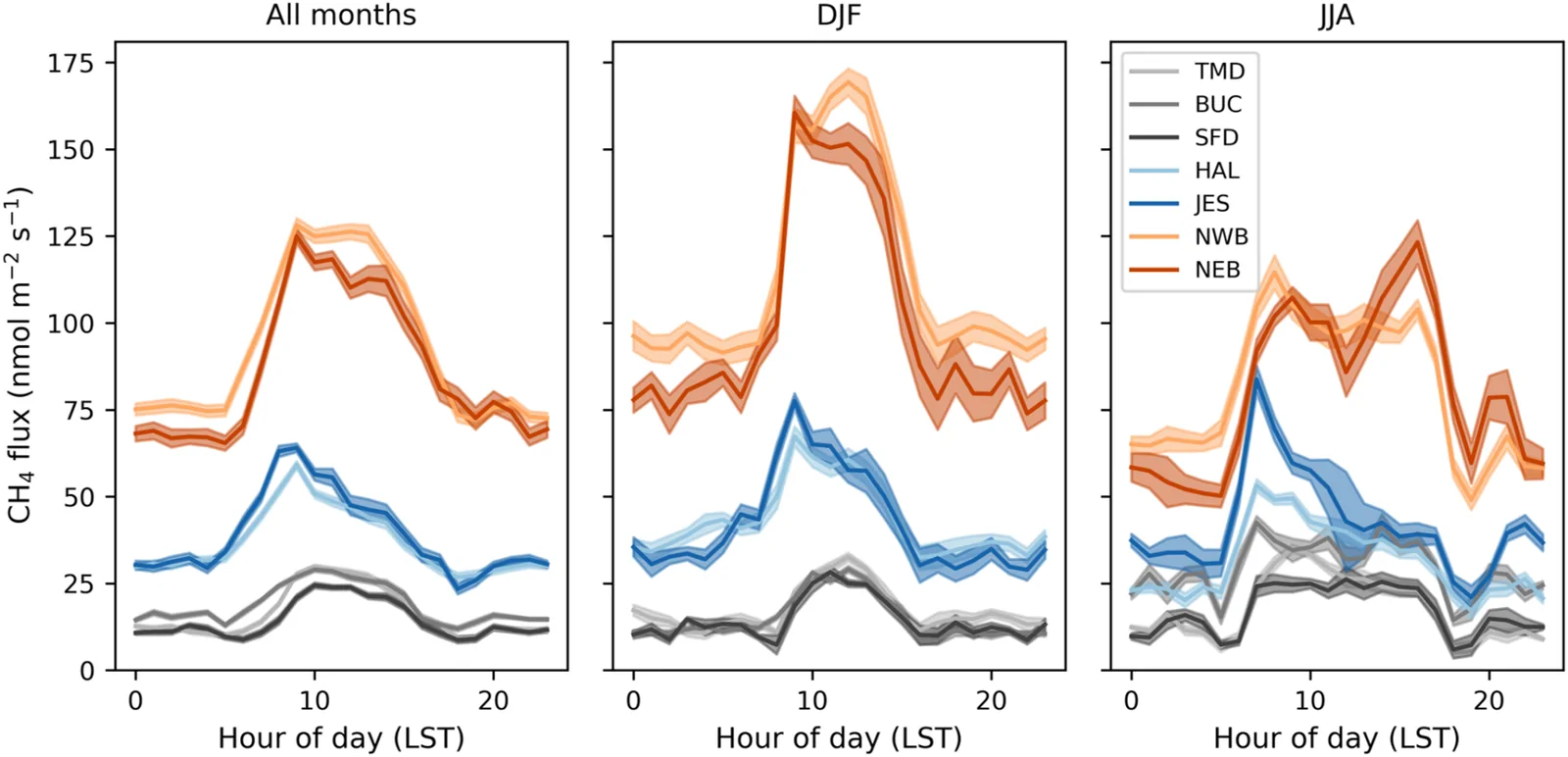
Average diurnal patterns of estimated CH_4_ fluxes at each measurement site for all months, winter months only (December, January, and February; DJF), and summer months only (June, July, and August; JJA). Each point represents the average of all data points from that hour of day for the entire data set, and the shaded region represents one standard error. Diurnal trends at rural sites (TMD, BUC, SFD) are shown in gray, suburban sites in blue (HAL, JES), and urban sites in orange (NWB, NEB). Temporal extents match those listed in Table 1.

#### Comparison to Natural Gas Usage Data

At NEB and NWB, the natural gas usage data and the estimated CH_4_ fluxes follow remarkably similar diurnal patterns to one another with lower magnitudes at night and peaks in the morning (Figure 6). The morning increase in estimated CH_4_ flux around 5–6 LST lags the morning increase in natural gas usage by about an hour on average (Figure 6), and the morning peak in estimated flux around 9 LST lags the peak in usage by an hour or more depending on site and season (Figure 7). The shape of the daily cycle also differs between the gas usage and estimated fluxes. The annual average daily cycle of CH_4_ flux estimates has a slower decrease from the morning peak back to the nighttime low compared to the natural gas usage (Figure 6), although this is not the case in the summer months (Figure 7). For both the natural gas usage and the flux estimates, the diurnal trend is broader in summer, staying higher for most of the day before decreasing back to a shorter-lived nighttime low (Figure 7).

**Figure 6:**
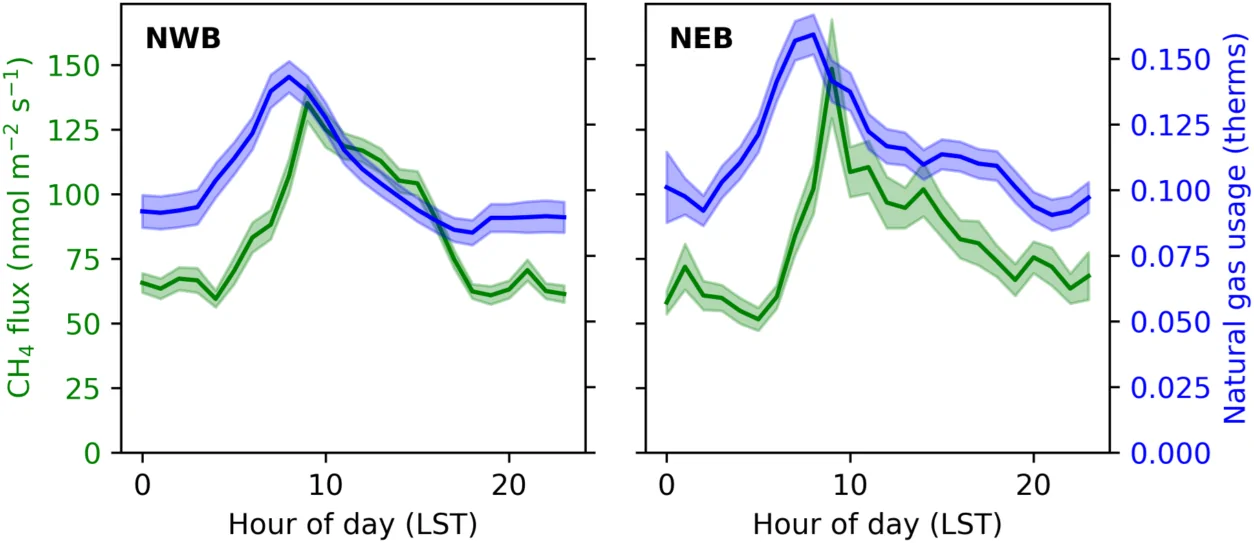
Average diurnal patterns of estimated CH_4_ fluxes and natural gas usage surrounding NWB and NEB for the year 2023. Each point represents the average of all data points from that hour of day, and the shaded region represents one standard error.

**Figure 7:**
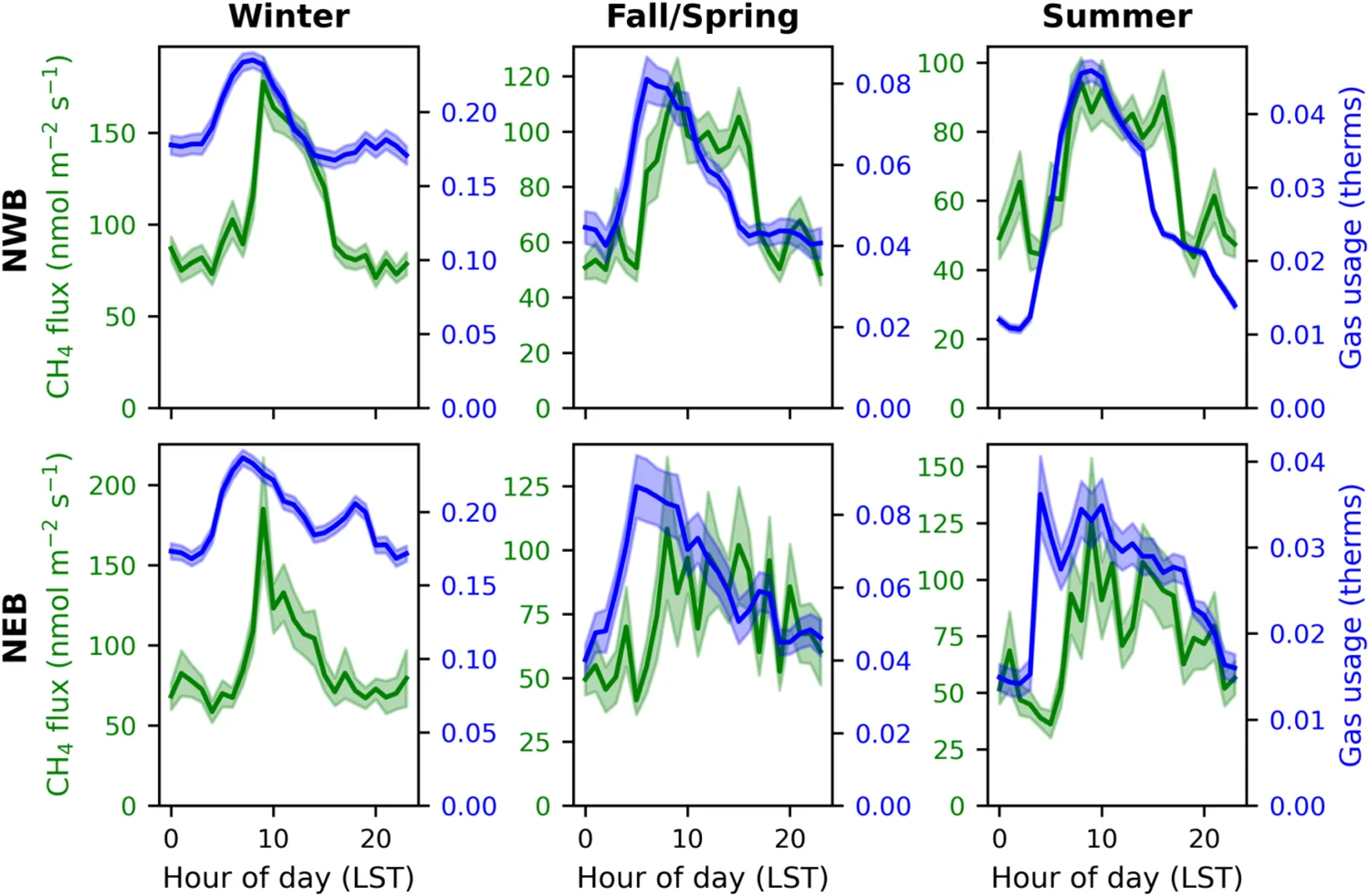
Average diurnal patterns of estimated CH_4_ fluxes and natural gas usage surrounding NWB and NEB divided into seasons for the year 2023. Winter includes January through March, November, and December. Fall/Spring includes April, May and October. Summer includes June through September. At NEB, 2 h of the year with anomalously high average gas usage values were removed (July 16th at 0 and 1 UTC, with average gas usages of 2.31 and 0.78 therms respectively) from this averaging to allow for visualization of the overall trend.

We expect that postmeter natural gas emissions are contributing to the diurnal trend in estimated CH_4_ fluxes. The usage data we present is measured at service points into buildings and residences, so a relationship between trends in this data and CH_4_ emissions could be related to emissions that occur after the point that the usage is measured. We expect that a substantial fraction of these postmeter emissions come from buildings and residences. Increased gas usage and emissions during daytime is likely related to use of gas appliances such as water heaters, furnaces, ovens and stoves, whose CH_4_ emissions spike when turned on or off.^20–22,58^ This trend is present in all seasons, with a larger amplitude in winter at the urban sites, suggesting that furnaces may play a role but may not entirely explain the diurnal cycle. We expect furnaces to be running continuously in winter, but furnace emissions could contribute to a diurnal cycle if users toggle the temperature in the morning. A higher frequency of switching other appliances such as water heaters and cooking appliances on and off during daylight hours is plausible, which would result in increased emissions during daytime. These appliances may also have a higher frequency of on/off pulses during daytime compared to furnaces, potentially resulting in their significant contribution to a daily cycle despite furnaces being responsible for more natural gas usage overall.^58^ While much of the footprint of the urban and suburban sites is residential, all four sites are also near commercial and/or industrial buildings. Postmeter emissions in these buildings, which likely have a different daily pattern of gas appliance usage, may also explain some of the diurnal cycle in gas usage and estimated CH_4_ emissions. In a restaurant, for example, one would expect significantly more on/off switches of cooking appliances during daytime hours compared to a residential building, potentially leading to more CH_4_ emissions.

We expect that leaks in the distribution pipeline do not depend on gas usage, but emissions further downstream may behave differently. Distribution pipelines are kept at a constant pressure regardless of usage, but pressure in the service lines may vary (personal communication, local gas company). Service line upkeep beyond a certain point downstream is the responsibility of the property owner rather than the gas company, so these parts of the service line and associated infrastructure may not subject to routine maintenance and upkeep the gas company provides for the distribution pipelines. Leaks or intentional venting at meter set assemblies have been reported in the literature with varying significance relative to overall fugitive natural gas emissions.^59–61^ Emissions from service lines, meter set assemblies, and associated infrastructure may contribute to higher daytime emissions alongside postmeter emissions, but it is difficult to say because these emissions are understudied. Future studies should investigate root cause of these emissions and their relationship with gas usage.

The hourly correlation between the estimated emissions and natural gas usage at both sites is low, but statistically significant, and increases slightly with a lag reaching a maximum at a lag of 2–3 h. The daily cycles of CH_4_ flux and gas usage at both sites have a statistically significant correlation that increases with increasing lag reaching a maximum at a lag of 2–3 h (Table 2).

The relationship between natural gas usage and potential postmeter emissions estimated from outdoor measurements is complicated by variable building air change rates (ACRs), which may explain the lag and affect correlations between the usage and emissions. Nazaroff^62^ reported a typical building ACR range of 0.2^–1^ h^–1^, suggesting that the highest correlation between usage data and estimated emissions we observe being at a 2- to 3 h lag could indeed be a result of indoor air residence time delaying the ventilation and detection of postmeter emissions by our measurements. This may also explain the low hourly and diurnal correlation, in some cases, between the gas usage and estimated emissions because postmeter emissions may not be emitted outside at the same rate as the true emission rate inside that is directly related to the natural gas usage. The ACR is dependent on time-varying factors including meteorology and human activity.^62^ Neglecting human activity, Malik^63^ reports a change in ACR of 0.1 h^–1^ for each 12 °F increase in indoor-outdoor temperature difference at low wind speeds or for each 2mph increase in wind (with relative angle of the wind to the building that has the largest impact on the ACR) at low indoor-outdoor temperature differences. Given this finding, one may expect higher winter and lower summer ACRs given Baltimore’s cold winters. However, the opposite pattern has been identified in similar climates, likely a result of increased window use during periods of smaller indoor-outdoor temperature differences, substantially increasing the ACR.^62^ For example, in Boston, Zota et al.^64^ found a winter median ACR of 0.49 h^–1^ and a summer median ACR of 0.85 h^–1^. Window use results in both seasonal and diurnal changes in ACR,^62^ which may further disconnect gas usage and our flux estimates, and may explain, for example, differences in the lag in the morning increase of estimated emissions compared to natural gas usage in summer compared to winter at NWB.

However, in-building residence time could only explain the lag between trends in gas usage and estimated emissions if a significant fraction of postmeter emissions come from indoors. Ventilation of exhaust outdoors is required for most gas appliances, with some exceptions including stoves.^65,66^ While many stoves are equipped with range hoods, these must be turned on by the user. Studies have shown that, in practice, range hoods are only used a small fraction of the time.^67–69^ Some range hoods do not vent outdoors, and some studies have found that, even when used, range hoods may not provide adequate ventilation.^69,70^ While we expect that cooking appliances contribute to the trends we see, we doubt that cooking appliances alone explain this lag. We are not aware of any studies monitoring the effectiveness of CH_4_ ventilation outdoors as it has no direct health impacts. Some studies have found an association between higher concentrations of indoor air pollutants and the presence of nonkitchen gas appliances^71,72^ indicating that at least some of their exhaust remains indoors. Sun et al.^72^ speculated backdraft, combustion spillage, and improper installation or insufficient maintenance of appliances as potential explanations for appliance exhaust remaining indoors. It is possible that such issues are indeed causing indoor emissions near our monitoring sites, or the lag between emissions and gas usage may be the result of another unknown factor. More information about the buildings in the footprint of our flux estimates would be needed to confirm or refute this hypothesis.

Other potential explanations for the low hourly or diurnal correlation in some cases include variety in the makeup of methane sources and spatial mismatch. Other methane sources, such as biogenic sources, do not depend on natural gas usage. Spatial mismatch between the footprint of our CH_4_ flux estimates may also decrease the correlation between the estimated fluxes and natural gas usage. Several studies have found that a small fraction of appliances may be responsible for a large fraction of emissions from appliances overall,^19,20,22^ which could potentially exacerbate the impacts of spatial mismatch on these comparisons.

Diurnal patterns with higher daytime CH_4_ emissions have been observed in other urban studies. Balashov et al.^23^ found CH_4_ emissions estimated using mole fraction data to be twice as high during daytime than at night in Indianapolis, IN. Using eddy covariance, similar diurnal patterns have been detected in London, UK,^30^ and Florence, Italy.^35^ The authors suggested postmeter natural gas emissions as a potential driver of this diurnal trend. A diurnal cycle in landfill emissions was also cited as a potential explanation,^73^ but we filtered out landfill plumes in this study. Schiferl et al.^74^ found evidence of a diurnal cycle in CH_4_ emissions in the New York City Metropolitan Area through a diurnal pattern present in the ratio of modeled and measured CH_4_ enhancements, where the modeled CH_4_ enhancements were based on an inventory in which emissions did not change by time of day. Schiferl et al. found a correlation between estimated CH_4_ and carbon monoxide (CO) emissions, suggesting that CO and CH_4_ may share incomplete combustion sources missing from the inventory. This supports the hypothesis that end-user emissions may drive a diurnal cycle in urban CH_4_ emissions.

Some urban studies, though, have found relatively flat diurnal trends in CH_4_ emissions^38,39^ or trends with different patterns than what we observe in our estimates.^36^ Hilland et al.^39^ found a smaller day-night difference in emissions in Zürich, Switzerland, using eddy covariance flux measurements where, in fact, CH_4_ emissions were higher on average at night than during the day. Stichaner et al.^38^ found a relatively flat trend using eddy covariance flux measurements in Innsbruck, Austria. The aforementioned diurnal trend in CH_4_ emissions detected with eddy covariance in London was present in some wind directions, while measurements from other wind directions showed a relatively flat trend.^30^ Stichaner et al.^38^ attribute most of the CH_4_ emissions they measured to postmeter natural gas emissions, and they note anecdotally that most gas cooking appliances in Europe have been replaced with electric. Even within postmeter emissions, the distribution of sources likely varies by location between and within cities, which could explain differences in diurnal trends between cities.

The presence of a smaller-amplitude diurnal trend at the rural sites, where we expect little natural gas infrastructure, suggests that other source sectors may contribute to the diurnal trend in estimated emissions. As speculated by Helfter et al.,^30^ higher daytime emissions could indicate a temperature-dependent biogenic source. This includes wetlands, which are present near BUC,^16^ or manmade water systems like wastewater conveyance systems,^24–26^ that are present near urban and suburban sites. Ren et al.^75^ reported that the major sources of CH_4_ emissions in the Baltimore/Washington region are natural gas and landfills, and that other biogenic sources make up a relatively small fraction of emissions, although we expect that the source makeup varies on a local scale.

This application of the flux-variance relationship in urban areas includes methodological uncertainties that should be considered, but it is doubtful that the diurnal pattern is entirely due to methodological error. One point of concern is the “widening” of the diurnal trend between summer and winter that may coincide with changes in atmospheric stability, which is considered in the flux-variance estimate. However, a similar change in the shape of the diurnal trend by season is observed in the natural gas usage data. This method has been evaluated in an urban environment in Indianapolis, IN, and did not show any evidence of presenting a diurnal cycle absent from the measured eddy covariance fluxes.^40^ This evaluation found a slightly larger day-night difference present in flux-variance estimates compared to measured eddy covariance fluxes, so it is possible that the day-night difference is exaggerated in our estimates. The presence of similar diurnal cycles in methane emissions in other urban areas from studies using different methodologies^23,30,35^ and the increased natural gas usage evident in the data we present here leads us to believe this is a real trend in CH_4_ emissions, although methodological uncertainty should be considered.

### Comparison to Karion et al.’s Inversion Prior and Posterior and Other Studies

The flux-variance estimates and Karion et al.’s posterior and prior fluxes show similar magnitudes (Table 3 and Figure 8) and both the flux-variance estimates and the inversion posterior identify a seasonal trend with higher winter emissions for urban sites (Figure 8). However, the inversion posterior shows a similar seasonal trend at HAL to that of the urban sites, which does not match the flatter trend at HAL in the flux-variance estimates. Average flux-variance estimates are within the range of the ensemble posterior fluxes at four out of the six sites and are within one standard deviation of the posterior flux mean at two out of the six sites (Table 3). Our estimated flux magnitudes and Karion et al.’s prior and posterior follow a similar ranking, with the two urban sites (NEB and NWB) having the largest flux magnitude and the rural sites (TMD and SFD) on the lower end, with the suburban site HAL in between, although Karion et al. have the suburban JES with magnitudes closer to the rural sites while our estimate is closer to HAL (Table 3 and Figure 8).

**Figure 8:**
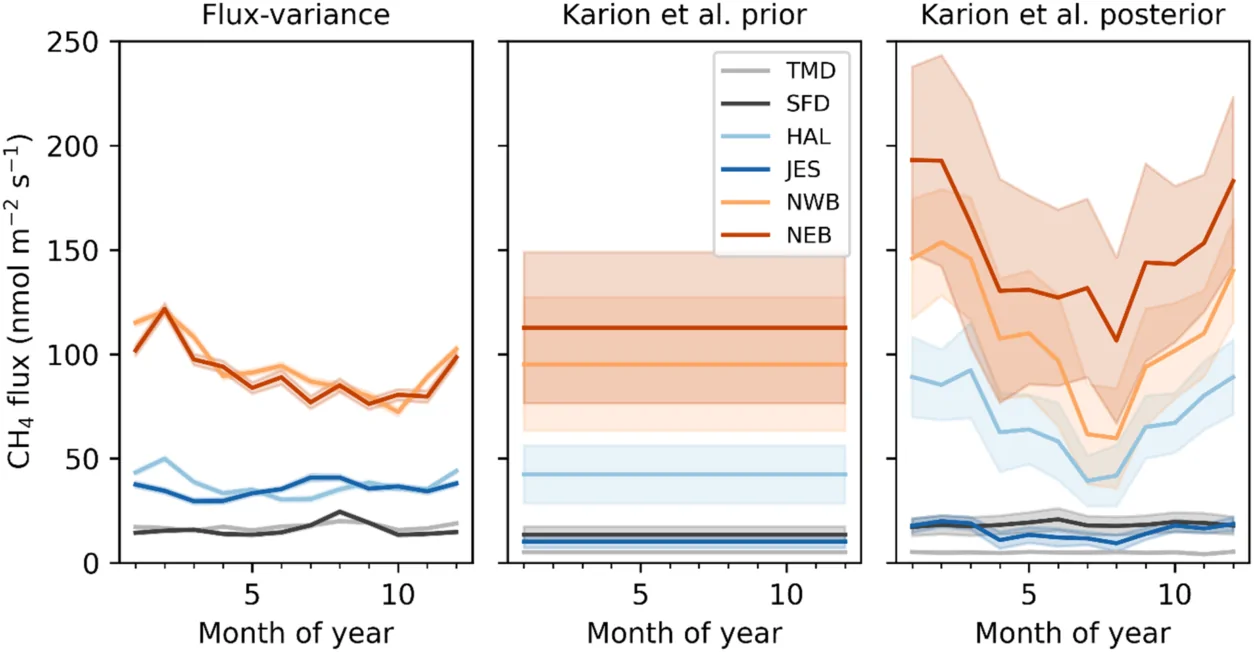
Mean seasonal trends for May 2017—December 2021 for the flux-variance estimates, Karion et al.’s inversion posterior ensemble mean, and Karion et al.’s inversion time-invariant prior ensemble mean.^53^ Seasonal trends at rural sites (TMD, BUC, SFD) are shown in gray, suburban sites in blue (HAL, JES), and urban sites in orange (NWB, NEB). The shaded regions around the flux-variance estimates represent the standard error calculated using statistical bootstrapping, around the prior ensemble means represent one standard deviation of the prior ensemble mean estimates from each ensemble member, and around the posterior ensemble means represent one standard deviation of the seasonal mean estimates from each ensemble member.

**Table 3:**
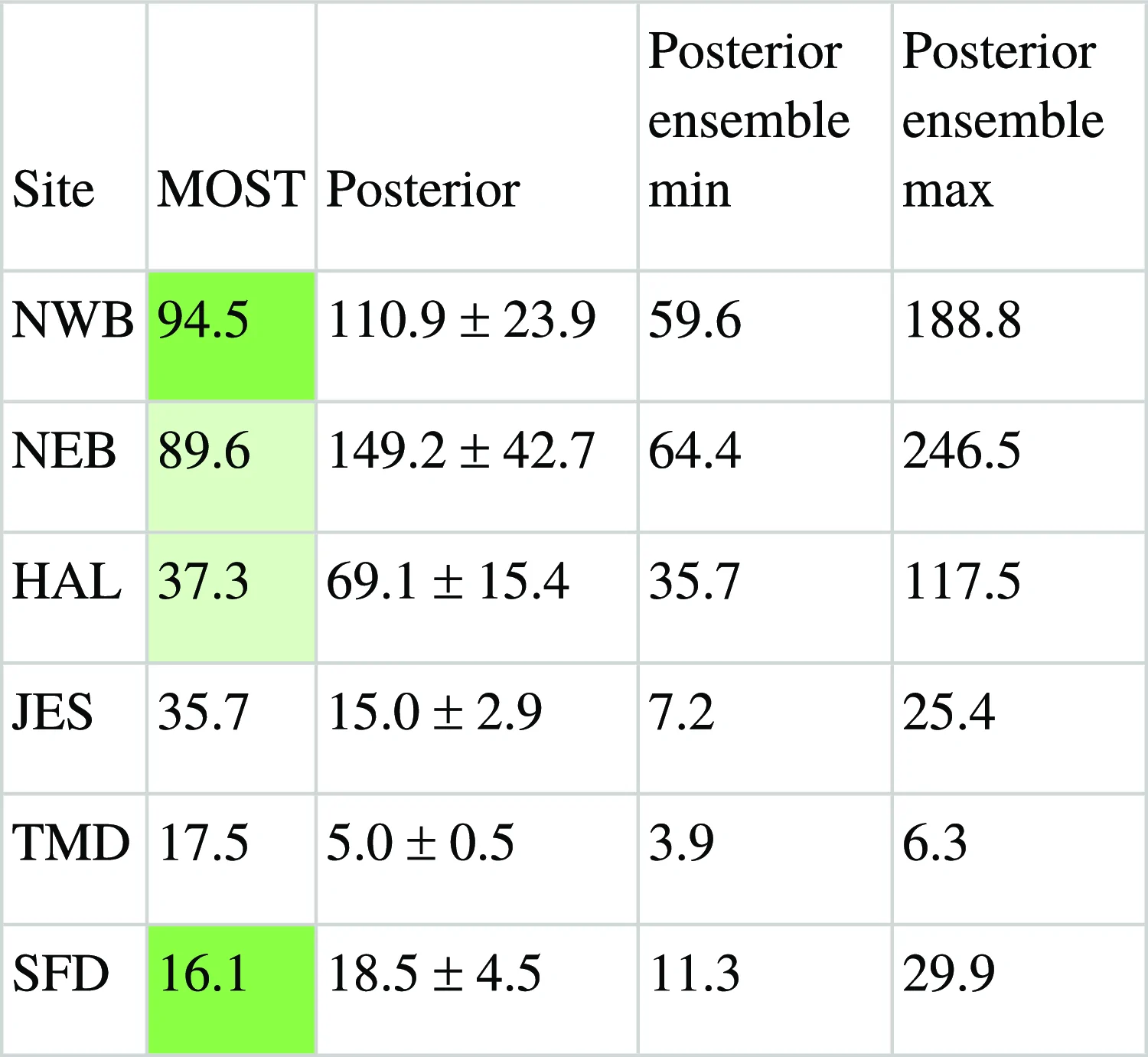
Comparison of Mean CH4 Fluxes Surrounding Each Site from May 2017 through December 2021 in nmol m^–2^ s^–1^ from This Study and from Karion et al.^16^ ^,^ ^a^

a MOST represents the flux-variance estimates presented in this paper. Posterior represents the mean plus or minus the standard deviations of the 60 posterior ensembles.^53^ MOST estimates that are between the posterior maximums and minimums are shaded light green, and MOST estimates that are within one standard deviation of the posterior ensemble mean are shaded dark green. BUC is not included because it is outside of the domain of Karion et al.’s estimate.

We reiterate that few tools are available to evaluate or coevaluate emissions estimates at this spatial scale. This coevaluation between local-scale estimates and a regional inversion estimate is, to our knowledge, the first of its kind. Karion et al.^16^ reported that the sectoral attribution of their estimate was dependent on the spatial allocation of sources in their prior, suggesting uncertainty in the spatial structure of their posterior emissions. Using individual grid cells from Karion et al.’s estimates to compare with our local-scale results is pushing the limits of the posterior’s expected accuracy and intended use.

The relative agreement between our estimated fluxes and those from Karion et al.’s^16^ prior and posterior increases our confidence in both estimates, particularly in the presence of urban-suburban differences in emissions magnitudes and trends and the pattern of elevated wintertime emissions around the urban sites. These similarities indicate convergence between independent methods. The spatial trend in Karion et al.’s prior is similar to that of their posterior, so we expect that the spatial trends in their posterior are driven primarily by the prior. This comparison shows similar spatial trends in emissions between the inventory-based prior and our flux-variance estimates. The seasonal trend in the inversion is driven by the atmospheric measurements. This comparison suggests that both the mole fraction enhancements and the variances detected similar seasonal trends in emissions. The difference in seasonal trend at HAL between the flux-variance and the inversion estimates could potentially be a result of spatial correlations assigned to measurement-based adjustments to the prior in the inversion setup. With HAL being closer to the urban center, posterior emissions in its surrounding grid cells could have been driven by measurements influenced by nearby emissions that have seasonal trends like those present at NWB or NEB. Other discrepancies between our results and Karion et al.’s could be due to errors in either or both flux estimation methods. The spatial matching of these estimates is very approximate, so differences between the footprint between the flux estimates and the area we considered from Karion et al.’s posterior could contribute to disagreement. Our sensitivity analysis provided in the Supporting Information revealed that assuming a larger footprint would not change our conclusions.

As in this study and by Karion et al., Huang et al.^76^ found higher wintertime emissions in the Baltimore/Washington metropolitan area (using some of the same tower-based measurements, ARL and HAL), although their domain is significantly larger than ours.

The magnitudes of our estimated fluxes in Baltimore are within the range of CH_4_ fluxes measured long-term with eddy covariance in other cities. The estimated CH_4_ fluxes averaged across the study period at the suburban sites are 37.7 nmol m^–2^ s^–1^ at HAL and 38.5 nmol m^–2^ s^–1^ at JES, similar to the mean flux of 34.4 nmol m^–2^ s^–1^ measured by Pawlak and Fortuniak^36^ at an urban site in Łódz, Poland. Measured CH_4_ fluxes lower in magnitude than our mean estimates at any of the urban or suburban sites in Baltimore have been reported at an urban site in Innsbruck, Austria^38^ (mean of 21.6 nmol m^–2^ s^–1^), an urban site in Zürich, Switzerland^39^ (mean of 13.3–13.5 nmol m^–2^ s^–1^), and a suburban site in Sakai, Japan^37^ (mean of 22 nmol m^–2^ s^–1^). The study in Japan also measured fluxes at an urban site,^37^ reporting higher fluxes there with a study period mean of 60 nmol m^–2^ s^–1^. Our mean flux estimates at the urban sites are high relative to the suburban sites in Baltimore and the aforementioned studies with a mean estimated flux of 95.8 nmol m^–2^ s^–1^ at NWB and 87.0 nmol m^–2^ s^–1^ at NEB. Higher eddy covariance CH_4_ fluxes have been reported in Florence, Italy,^35^ at a mean of 136.9 nmol m^–2^ s^–1^ and in London, England,^30^ at a mean of 145.0 nmol m^–2^ s^–1^. Time domains differ between these studies, and even between the sites within our study, so these comparisons are approximate. We also reiterate that there is some degree of methodological uncertainty in the magnitude of the flux-variance estimates as described in the Methods and Data section.

### Implications and Recommendations for Future Work

The seasonal trend in and larger magnitude of CH_4_ emissions observed at urban sites in Baltimore, but not at suburban or rural sites, suggests that seasonal and spatial trends in urban CH_4_ emissions may be driven by leaks in natural gas distribution systems. We expect that the presence of a seasonal trend depends on gas distribution pipeline material and that the trend in emissions from distribution system leaks is dictated more by leak maintenance schedules than natural gas usage. Previous studies suggest that the significance of this source varies between cities due to differences in pipeline material and age,^9,15,55^ and we add that the within-city variability of this source is also substantial.

We also identified a diurnal trend in CH_4_ emissions that we expect is driven by postmeter natural gas emissions and potentially some biogenic emissions sources such as wastewater conveyance systems. The magnitude of the day-night difference suggests that postmeter natural gas emissions are an important source of methane in urban areas and should be considered in emissions quantification efforts. Future studies using eddy covariance to measure urban CH_4_ flux could increase our confidence in the presence of this diurnal trend, and quantifying diurnal trends of tracers such as ethane or CO could help disentangle sources of this trend and potentially confirm that natural gas emissions are a significant driver and whether incomplete combustion plays an important role. Further evaluation of diurnal trends in different urban environments could improve our understanding of their sources. Understanding why there is a lag between gas usage and estimated emissions and whether that lag is present in other cities is an area for further research.

We reiterate that the possibility of a diurnal pattern in CH_4_ emissions should be considered for urban emissions estimation methods that rely on afternoon observations, as noted by Schiferl et al.^74^ It is not uncommon to use only afternoon observations to constrain inverse emissions estimates (e.g., Yadav et al.,^17^ Sargent et al.^6^) to minimize model transport error. If daytime emissions are higher, assuming a flat diurnal trend and using only afternoon observations may result in a high bias in estimated CH_4_ emissions.

These results provide a conceptual foundation for improving the bottom-up modeling of urban CH_4_ emissions. Flux-variance relationships provide a sound approach to inferring spatial and temporal patterns in local-scale methane emissions, which could be applied to existing urban atmospheric methane mole fraction measurements in different cities. These flux inference methods can be used to develop and test emissions inventories. In Baltimore, we find evidence of a relationship between urban CH_4_ emissions and natural gas usage, pipe material, and leak maintenance schedules (time of year). Future work could develop a bottom-up emissions model that links these processes quantitatively with CH_4_ emissions from our atmospheric observations. This model could then be tested across a variety of urban environments to improve our mechanistic understanding of the emissions of methane from urban environments.

## Supplementary Material



## Data Availability

The methane mole fraction data used for this study is openly available in the NIST Public Data Repository at 10.18434/mds2-3012. The spatially averaged natural gas usage data used for this study is available at 10.57931/3376263. The ASOS station data used for this study is openly available through the Iowa Environmental Mesonet at https://mesonet.agron.iastate.edu/request/download.phtml?network=MD_ASOS (stations BWI, CGE, NHK, FDK, RSP) and https://mesonet.agron.iastate.edu/request/download.phtml?network=VA_ASOS (stations RMN, DAA).

## Notes

Data processing information, Pearson correlation coefficients, and a sensitivity test for spatial uncertainty for the comparison to Karion et al. (PDF)

## §

School of Environment and Sustainability, University of Cincinnati, Cincinnati, Ohio 45219, United States
