# Supplementary material for: Emissions from Gas Distribution and Postmeter Leaks May Drive Seasonal and Diurnal Trends in Methane Emissions in Baltimore
Source: Environ Sci Technol. 2026 Sep 2;60(36):25413–25. doi: 10.1021/acs.est.6c03452 (PMC13585070; doi:10.1021/acs.est.6c03452)
Supplement: Supplementary file 1 [file es6c03452_si_001.pdf]

Supporting information for *Emissions from gas distribution and postmeter leaks may drive seasonal and diurnal trends in methane emissions in Baltimore*

Helen C. R. Kenion[α,†], Kenneth J. Davis[α,β], Natasha L. Miles[α]*

[α]Department of Meteorology and Atmospheric Science, The Pennsylvania State University, University Park, Pennsylvania 16802, United States

[β]Earth and Environmental Systems Institute, The Pennsylvania State University, University Park, Pennsylvania 16802, United States

Data processing for mole fraction standard deviation

The CRDS measures the CH_4 mole fraction at approximately 2-3 second intervals. The data output includes both 1-minute statistics of the mole fraction measurements, including mean, standard deviation, and count of each minute. In a separate file, the hourly statistics of the 1-minute means, including the average and standard deviations of the 1-minute means for each hour, are also reported. For the flux-variance calculation, it is necessary to use the standard deviation of the raw 2-4 second measurements to include contributions to the standard deviation from all measured frequencies.

However, the raw data (~2-3 second intervals) is not reported. Thus, we calculated the raw standard deviation using the 1-minute statistics of the raw data. We followed the methods described in detail in the PDF titled Standard_Deviation_Calculation.pdf published alongside the mole fraction data we used[1], which allowed us to unambiguously calculate the raw standard deviation, which we then used in our flux estimate calculations.

We used the standard deviation of the mole fraction measurements from a given inlet to estimate fluxes as described in the methods section, and we need to use the standard deviation calculated from continuous measurements. The 40-minute/20-minute sampling cycle does not necessarily align with the hour, meaning the hourly statistics reported by Karion et al. may include parts of multiple cycles instead of one continuous cycle, another reason we did not use the hourly data files. To avoid using standard deviations calculated from multiple separate cycles, we calculated the standard deviation of each continuous measurement segment (from the 1-minute statistics) rather than using a changing of hour to separate each flux estimate.

A measurement frequency of 10 Hz for a duration of 30 minutes is generally considered sufficient to represent the full power spectrum[2], meaning statistics from measurements collected at a lower frequency for a shorter duration will underestimate the flux. We use measurements collected every 2-3 seconds (≤0.5Hz, resulting in spectral loss in the high frequencies) and sometimes for durations less than 30 minutes (spectral loss at lower frequencies). To mitigate spectral loss that would result in flux underestimation, we applied spectral correction factors developed by Kenion et al.3 to the standard deviations based on sampling time and frequency.

An example of mole fraction data before processing is shown in Figure S1. This 2-hour window at NWB has two 20-minute periods of continuous sampling (sampling times vary by site and sometimes within a site’s dataset). For a given 20-minute period, we have the mean, standard deviation, and count of data points (not shown) for each minute of sampling. For a given period of continuous sampling, we

used these 1-minute statistics within the period to calculate the standard deviation of the raw (2-3 second interval) data for the whole continuous sampling period (20 minutes in this example) following the methodology described by Karion et al.[1]. This raw standard deviation is what we used to calculate our flux-variance estimates. The data shown in Figure S1 would be used to produce two flux estimates, one for each period of continuous sampling. Dividing up the data by hour (i.e., 19:00 – 20:00 for one flux estimate) would not be appropriate because it would divide the data into smaller sampling periods than necessary and, in this case, lump together two separate sampling periods over 30 minutes apart.

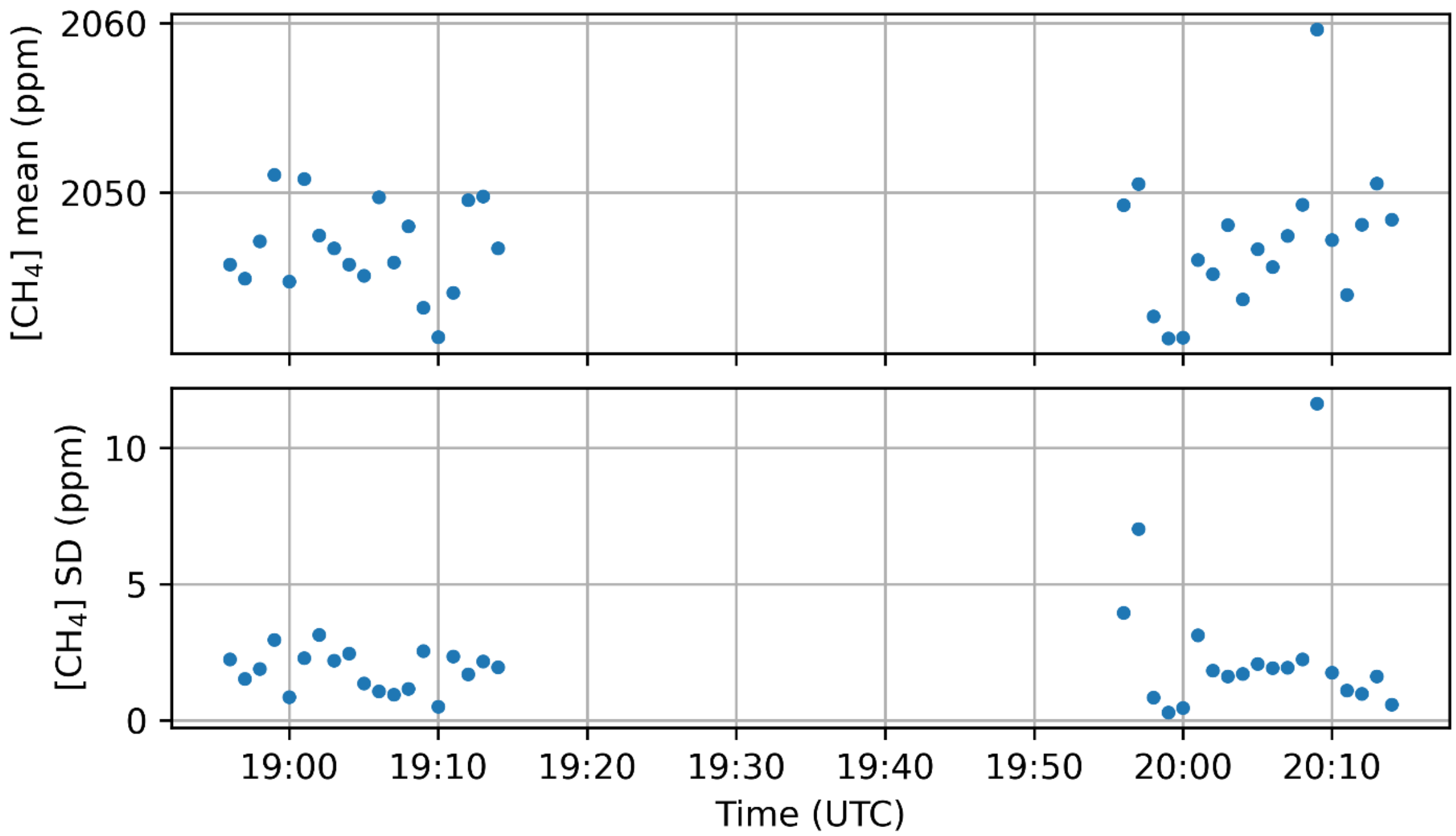

Figure S1. CH_4 mole fraction data from NWB before processing. The upper plot shows the reported mean of each minute, and the lower plot shows the reported standard deviation of each minute.

Pearson correlation between natural gas usage and estimate fluxes

Table S1. Pearson r values between natural gas usage data and flux estimates at NEB and NWB for the year 2023.[a]

	**No lag**			**1 hour lag**		**2 hour lag**		**3 hour lag**	
	**Hourly**	**Diurnal**	**Monthly**	**Hourly**	**Diurnal**	**Hourly**	**Diurnal**	**Hourly**	**Diurnal**
**NWB**	0.25*	0.64*	0.87*	0.27*	0.80*	0.28*	0.91*	0.28*	0.95*
**NEB**	0.019	0.52*	0.38	0.027	0.67*	0.038*	0.81*	0.047*	0.90*

Spatial uncertainty in comparison to Karion et al. prior and posterior

For the comparison of our estimated fluxes with Karion et al.'s inversion prior and posterior[4,5], we assumed a uniform footprint extent of 1km from the measurement location, resulting in including grid cells whose center points lie within 1.8km of the measurement site as described in the methods. We performed a sensitivity analysis by performing the same comparison, but with an assumed uniform footprint extent of 2km from the measurement location, meaning we included grid cells whose center points lie within 2.8km of the measurement site. While we expect the bulk of the "true" physical footprint is weighted closer to the site than 2.8km, this test helps us understand how much changes in our assumed footprint would affect our results.

Extending the assumed footprint affects the results but does not change our overarching conclusions. Changing the footprint size affects the prior and posterior averages at the urban sites the most and has little impact on the suburban and rural sites (Figure S2, Table S2). The ranking of emissions by site remains the same as with the 1km footprint. The 2km footprint results in higher prior and posterior average emissions at NWB and lower at NEB compared to the 1km footprint, resulting in the magnitude

[a] We also present hourly and diurnal correlations with the natural gas usage data lagged by 1-3 hours. "*" indicates the correlation is statistically significant at 95% confidence. This table is the same as Table 2 in the main text, except with Pearson r instead of Spearman r.

of emissions at NEB to be closer to that of the suburban site HAL than the other urban site NWB. The change in footprint size has little affect on general seasonal trends at all sites.

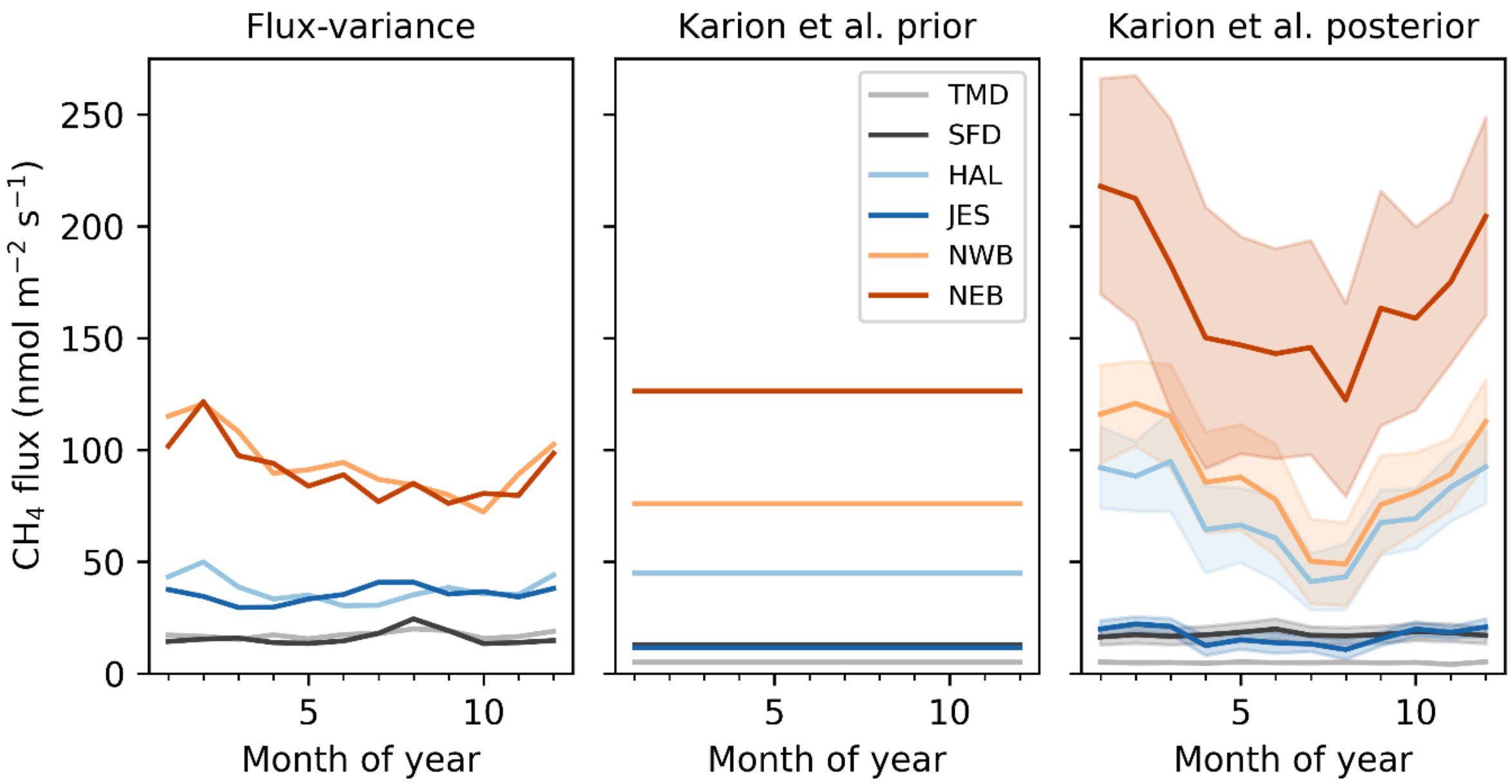

Figure S2. The same as Figure 7 in the main text, except assuming footprint of 2km for the flux estimates when selecting grid cells from Karion et al.'s prior and posterior to average for the comparison to each site.

Table S2. Comparison of mean CH_4 fluxes surrounding each site from May 2017 through December 2021 in nmol m^{-2} s^{-1} from this study and from Karion et al.[4,5] [b] assuming a 2km footprint extent.

---

[b] This is the same as Table 3 in the main text, but using a 2km instead of a 1km assumed footprint extent. MOST represents the flux-variance estimates presented in this paper. Posterior represents the mean plus or minus the standard deviations of the 60 posterior ensembles. MOST estimates that are between the posterior maximums and minimums are shaded light green, and MOST estimates that are

Site	MOST	Posterior	Posterior ensemble min	Posterior ensemble max
NWB	94.5	88.7 ± 18.8	47.2	148.1
NEB	89.6	167.9 ± 47.0	72.7	281.9
HAL	37.3	71.6 ± 15.0	37.8	119.5
JES	35.7	16.8 ± 3.2	8.3	28.8
TMD	17.5	5.0 ± 0.5	3.8	6.2
SFD	16.1	17.7 ± 3.8	10.8	27.8

Supplement References

(1) Karion, A.; DiGangi, E.; Prinzivalli, S.; Draper, C.; Baldelli, S.; Fain, C.; Biggs, B.; Stock, M.; Michalak, B.; Salameh, P.; Kim, J.; Luecker, T.; Whetstone, J. Observations of Carbon Dioxide (CO2), Methane (CH4), and Carbon Monoxide (CO) Mole Fractions from the NIST Northeast Corridor Urban Testbed. *NIST Public Data Repository*, 2025. https://doi.org/10.18434/mds2-3012.

(2) Foken, T. Experimental Methods for Estimating the Fluxes of Energy and Matter. In *Micrometeorology*; Foken, T., Ed.; Springer Berlin Heidelberg: Berlin, Heidelberg, 2017; pp 143–205. https://doi.org/10.1007/978-3-642-25440-6_4.

(3) Kenion, H. C. R.; Davis, K. J.; Miles, N. L.; Monteiro, V. C.; Richardson, S. J.; Horne, J. P. Estimation of Urban Greenhouse Gas Fluxes from Mole Fraction Measurements Using Monin-Obukhov Similarity Theory. *Journal of Atmospheric and Oceanic Technology* **2024**, *41* (9), 833–846. https://doi.org/10.1175/JTECH-D-23.

(4) Karion, A.; Ghosh, S.; Lopez-Coto, I.; Mueller, K.; Gourdji, S.; Pitt, J.; Whetstone, J. Methane Emissions Show Recent Decline but Strong Seasonality in Two US Northeastern Cities. *Environmental Science and Technology* **2023**, *57* (48), 19565–19574. https://doi.org/10.1021/acs.est.3c05050.

(5) Karion, A.; Ghosh, S.; Lopez-Coto, I.; Mueller, K.; Gourdji, S.; Pitt, J.; Whetstone, J. Methane Emissions Estimates and Related Data Sets for Washington DC and Baltimore, MD Urban Areas. *NIST Public Data Repository*, 2025. https://doi.org/10.18434/mds2-3720.

within one standard deviation of the posterior ensemble mean are shaded dark green. BUC is not included because it is outside of the domain of Karion et al.'s estimate.
